# ZC3H12D upregulation in head and neck squamous cell carcinoma: a potential prognostic biomarker associated with immune infiltration

**DOI:** 10.1590/1414-431X2024e14227

**Published:** 2025-02-03

**Authors:** Mingyu Zhao, Wenkai Huang, Xu Huang, Feihan Gu, Lin Yang, Yuanyin Wang, Ran Chen

**Affiliations:** 1 College & Hospital of Stomatology, Anhui Medical University, Key Lab. of Oral Diseases Research of Anhui Province, Shushan District, Hefei, China

**Keywords:** ZC3H12D, Immune infiltration, Prognosis, HNSCC, TCGA

## Abstract

Head and neck squamous cell carcinoma (HNSCC) is a common malignant tumor that poses a major hazard to people's health. ZC3H12D, which belongs to the family of CCCH-type zinc finger-containing proteins, is a negative regulator with a key function in immune modulation. However, it is still unclear how ZC3H12D affects the immune infiltration and prognosis of HNSCC. In this study, the data obtained from various databases were used to assess ZC3H12D expression in HNSCC and in various tumors under the HNSCC classification. The association between clinical features and ZC3H12D expression in HNSCC was evaluated using the UALCAN database. Additionally, a ROC curve was employed to analyze the diagnostic value of ZC3H12D. The effect of ZC3H12D on prognosis was assessed using Kaplan-Meier curves, Cox analysis, and the nomogram model. Gene Set Enrichment Analysis, Gene Ontology, and Kyoto Encyclopedia of Genes and Genomes enrichment analyses were employed to investigate the underlying role of ZC3H12D in HNSCC. The association between ZC3H12D expression and the tumor microenvironment and immune checkpoints were investigated by TIMER2 and Tumor Immune Single Cell Hub 2 databases and various packages in R. The findings demonstrated a significant up-regulation of ZC3H12D expression in HNSCC, while ZC3H12D expression was found to be associated with clinical parameters. Our study also demonstrated that ZC3H12D could act as a potential prognostic biomarker for HNSCC, especially oral squamous cell carcinoma. Additional analyses have shown that ZC3H12D was associated with common immune checkpoint genes and may be related to immune infiltration in HNSCC.

## Introduction

Head and neck squamous cell carcinoma (HNSCC) is a common malignant tumor that poses a major hazard to people's health (1). Although surgery, chemotherapy, and radiotherapy keep improving, the overall survival rate of HNSCC patients is still low (2). Additionally, the capacity to evaluate patient prognosis and clinical treatment using conventional staging methods based on pathological features is somewhat limited (3). Immunotherapy has been shown to significantly increase quality of life and overall survival in patients with HNSCC, particularly in those who are positive for PD-1, HPV, or PD-L1 (4,5). Hence, for the prognosis of HNSCC and the advancement of immunotherapy against HNSCC, the identification of reliable biomarkers is crucial.

ZC3H12D, also referred to as p34 and TFL, belongs to the family of CCCH-type zinc finger-containing proteins, along with ZC3H12C, ZC3H12B, and ZC3H12A (6,7). ZC3H12D is enriched in inflamed and lymphoid tissues like lung, lymph node, and spleen. ZC3H12D overexpression markedly suppressed TLR-induced NF-κB, ERK, and JNK activation in macrophages (8). It has been demonstrated that ZC3H12D negatively regulates Toll-like receptor signaling and targets the mRNA of c-fos and NF-κB as well as cytokines to inhibit chronic inflammation and initial inflammation storm (9). Recently, the relationship between ZC3H12D and tumors has attracted the attention of an increasing number of researchers. A study showed that in HNSCC patients, the ZC3H12D expression was positively correlated with the levels of PD-1 and PD-L1, and the methylation level of the ZC3H12D promoter was lower than that of normal patients (10). Also, ZC3H12D is demonstrated to be highly expressed in human endometrial carcinomas but not or only weakly expressed in normal tissues, and patients with endometrial cancer with high expression of ZC3H12D have a better prognosis (11). Furthermore, ZC3H12D was shown to be specifically upregulated in lung adenocarcinoma (LUAD), and increased ZC3H12D expression is associated with the increase in B cells and a better prognosis of LUAD (12). Nevertheless, the underlying mechanisms and effect of ZC3H12D in HNSCC are not fully understood.

The aim of the present study was to explore ZC3H12D's prognostic value and its effect on immune infiltration in HNSCC by using comprehensive bioinformatics analysis.

## Material and Methods

### Data acquisition and gene expression analysis

The RNA-sequencing data and corresponding clinical information for 44 normal and 497 HNSCC samples were collected from The Cancer Genome Atlas database (TCGA). Based on extensive genome sequencing, 33 cancer types are currently represented in TCGA database, which is the largest available database of information on cancer genetics, and transcriptomic, genomic, proteomic, epigenetic, and other omics data. We compared the expression of ZC3H12A, ZC3H12B, ZC3H12C, and ZC3H12D in normal and tumor samples from TCGA database. In Supplementary Table S1, the ZC3H12D expression and complete clinical information of 497 patients with HNSCC are listed. The dataset GSE30784, GSE127165, and GSE12452 were retrieved from the Gene Expression Omnibus (GEO) database. The GSE30784 dataset includes data from 45 normal samples and 167 oral squamous cell carcinoma (OSCC) samples, and the GSE127165 dataset includes data from 57 laryngeal squamous cell carcinoma (LSCC) samples and 57 paired adjacent normal samples. The GSE12452 dataset includes data from 31 nasopharyngeal carcinoma (NPC) samples and 10 normal samples. Head and neck squamous cell carcinoma (HNSCC) consists primarily of cancers of the oral cavity, larynx, and pharynx (13). We assessed the differential expression of ZC3H12D between OSCC, LSCC, pharyngeal carcinoma, and normal tissues by the datasets from the GEO and TCGA databases. The Human Protein Atlas (HPA) was employed to obtain the ZC3H12D expression data that were obtained by immunohistochemical staining studies (14). Using the UALCAN database, a comprehensive analysis of ZC3H12D expression in various clinical subgroups classified according to tumor grade, individual cancer stage, age, race, gender, TP53 mutation status, nodal metastasis status, and HPV infection status was performed (15). The AlphaFold protein structure database was utilized to predict the protein structure of ZC3H12D (16). ZC3H12D chromosomal location was visualized with the “RCircos” package in R. The genetic alteration features as well as mutation sites of ZC3H12D were identified utilizing the cBioPortal database (17).

### Analysis of prognostic value

Using receiver operating characteristic (ROC) curve, ZC3H12D's diagnostic value was assessed, and the diagnostic value was determined as the area under the curve (AUC). ROC analysis was performed with the “pROC” package in R. The optimal separation method was employed to split the patients into ZC3H12D low-expression group and high-expression group before the Kaplan-Meier (K-M) approach was applied to construct the survival curve. K-M survival analysis was conducted using the “survminer” and “survival” packages in R (v.4.2.0). The K-M survival curves of ZC3H12D were also created with Gene Expression Profiling Interactive Analysis 2 (GEPIA2) database (18). According to the clinical data obtained from the GSE41613 dataset in GEO, the survival role of ZC3H12D in OSCC was further investigated. In order to determine whether ZC3H12D could be employed as a prognostic factor independent of grade, T stages, age, N stages, and gender, univariate and multivariate Cox regression analyses were carried out. Additionally, the nomogram model was constructed on the basis of multivariate Cox analysis through the “rms” package in R software. Furthermore, the model was evaluated by calibration curves.

### Identification of ZC3H12D-related genes and functional enrichment analysis

ZC3H12D co-expression analysis was performed using the TCGA HNSCC cohort, with LinkedOmics website (19). In the correlation analysis, co-expressed genes were identified as those with adjusted false discovery rate (FDR) <0.05 and |cor| >0.4 in the Pearson correlation test (Supplementary Table S2). According to ZC3H12D's median expression value, the HNSCC samples from TCGA database were split into two groups: high-expression group and low-expression group. Using Padj <0.05 and |logFC| >1.0 as thresholds, the “DESeq2” package in R was employed to identify the differentially expressed genes between low-expression and high-expression groups. Using the “clusterProfiler” package in R software, Gene Set Enrichment Analysis (GSEA) was performed on the differentially expressed genes, while Gene Ontology (GO) and Kyoto Encyclopedia of Genes and Genomes (KEGG) enrichment analyses were conducted on the co-expressed genes of ZC3H12D. A controlled, dynamic, and comprehensive source for functional genomics was offered through the Gene Ontology Consortium (20), which included cellular component (CC), biological process (BP), and molecular function (MF).

### Immune infiltration analysis

Using established methods such QUANTISEQ, MCPCOUNTER, EPIC, XCELL, TIMER, and CIBERSORT-ABS, the degree of immune infiltration in HNSCC patients was ascertained; the correlation between immune cell infiltration and ZC3H12D expression was then assessed (21-26). Each HNSCC sample's tumor microenvironment (TME) status was assessed by the “ESTIMATE” package in R, and the outcomes were displayed as ESTIMATE/stromal/immune scores (27). The scores of the high and low ZC3H12D expression groups were contrasted using the “ggpubr” package in R software to show the differences. Additionally, Single-sample Gene Set Enrichment Analysis (ssGSEA) was used to determine differences in immunity function between these groups. Using K-M survival analysis from the TIMER database, the effect of ZC3H12D expression and immune cell infiltration on overall survival of HNSCC patients was examined. We also performed prognostic analysis of HNSCC patients with different ZC3H12D expression and immune cell infiltration utilizing the TIMER2 database (28). As a resource of scRNA-seq data from mouse and human tumors, the Tumor Immune Single Cell Hub 2 (TISCH2) is able to comprehensively assess the gene expression in the TME in various tumor types (29). ZC3H12D expression status in various cell types in the TME was investigated based on two datasets (GSE150321 and GSE150430) by the TISCH2 (http://tisch.comp-genomics.org/) database.

### Analysis of immune checkpoint genes

We investigated the association between ZC3H12D expression and the main immune checkpoint genes (including SIGLEC15, PDCD1LG2, HAVCR2, CTLA4, PDCD1, CD274, TIGIT, and LAG3) using TIMER2 in light of the crucial role immune checkpoint inhibitors perform in immune therapy. In addition, we compared the expression of the major immune checkpoint genes between the HNSCC patients with high and low ZC3H12D expression.

### Statistical analysis

Wilcoxon rank-sum test was employed to assess how differently ZC3H12D was expressed in normal and HNSCC samples. Pearson's chi-squared test was employed to evaluate the association between the clinicopathological variables and the mRNA expression of ZC3H12D. Multivariate and univariate Cox regression analyses were performed. Spearman correlation analysis was applied to calculate the correlation between immune infiltration cell scores and ZC3H12D expression. The differences in the immune infiltration cell scores and TME scores between the groups with low and high ZC3H12D expression were evaluated by the Wilcoxon rank-sum test. R software (v4.2.0) was employed to conduct all of the aforementioned statistical analyses. Statistical significance was set at P<0.05 unless otherwise specified.

## Results

### Increased ZC3H12D expression in HNSCC

**Figure 1:**
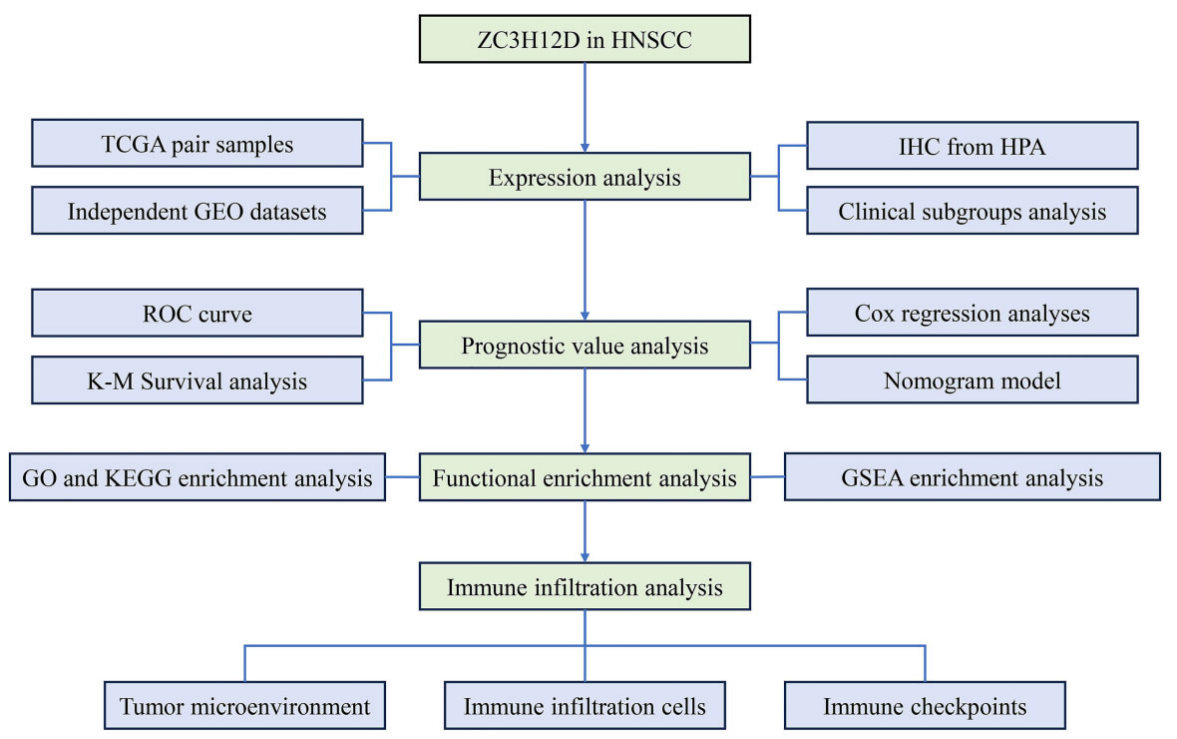
Workflow diagram for the present research. HNSCC: head and neck squamous cell carcinoma; TCGA: The Cancer Genome Atlas; GEO: Gene Expression Omnibus; ROC: receiver operating characteristic; K-M: Kaplan-Meier; GO: Gene Ontology; KEGG: Kyoto Encyclopedia of Genes and Genomes; IHC: immunohistochemistry; HPA: Human Protein Atlas; GSEA: Gene Set Enrichment Analysis.

**Figure 2:**
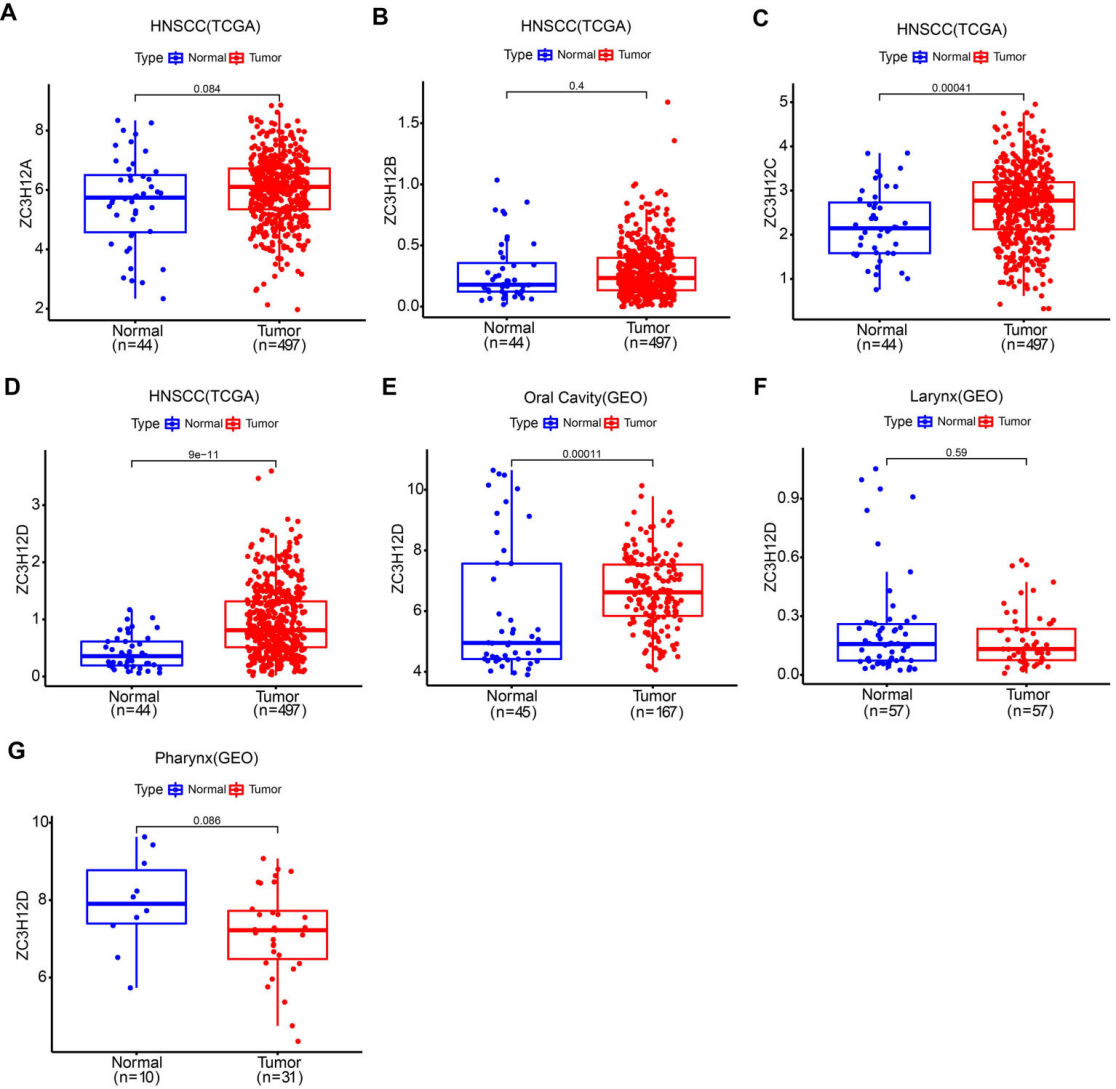
Gene expression analysis. **A**-**D**, ZC3H12A, ZC3H12B, ZC3H12C, and ZC3H12D expression analysis in normal and head and neck squamous cell carcinoma (HNSCC) samples from the TCGA database. **E**, Expression of ZC3H12D in normal and oral squamous cell carcinoma (OSCC) samples based on data from GSE30784. **F**, Expression of ZC3H12D in normal and laryngeal squamous cell carcinoma (LSCC) samples based on data from GSE127165. **G**, Expression of ZC3H12D in normal and pharyngeal carcinoma samples based on data from GSE12452. Data are reported as median and IQR. Wilcoxon rank-sum test.

Figure 1 displays the flow chart for the experiments conducted in this research. Using data retrieved from TCGA database, analysis of ZC3H12A, ZC3H12B, ZC3H12C and ZC3H12D expression in HNSCC and normal samples was performed (Figure 2A-D). The results showed that the difference in expression of ZC3H12A and ZC3H12B between HNSCC and normal samples was not statistically significant, and the expression of ZC3H12C and ZC3H12D was higher in HNSCC samples compared to normal samples. Furthermore, the expression difference of ZC3H12D was more significant compared with ZC3H12C. Therefore, ZC3H12D was selected as the target gene for further research. In addition, ZC3H12D overexpression in OSCC samples was demonstrated in the independent dataset from GEO (accession number: GSE30784) (Figure 2E). There was no statistically significant difference in ZC3H12D expression between LSCC as well as pharyngeal carcinoma and normal tissues, according to the GEO dataset (accession number: GSE127165 and GSE12452) (Figure 2F and G). Data from the TCGA database indicated that ZC3H12D was overexpressed in OSCC as well as LSCC samples compared to normal samples (Supplementary Figure S1A and B).

**Figure 3:**
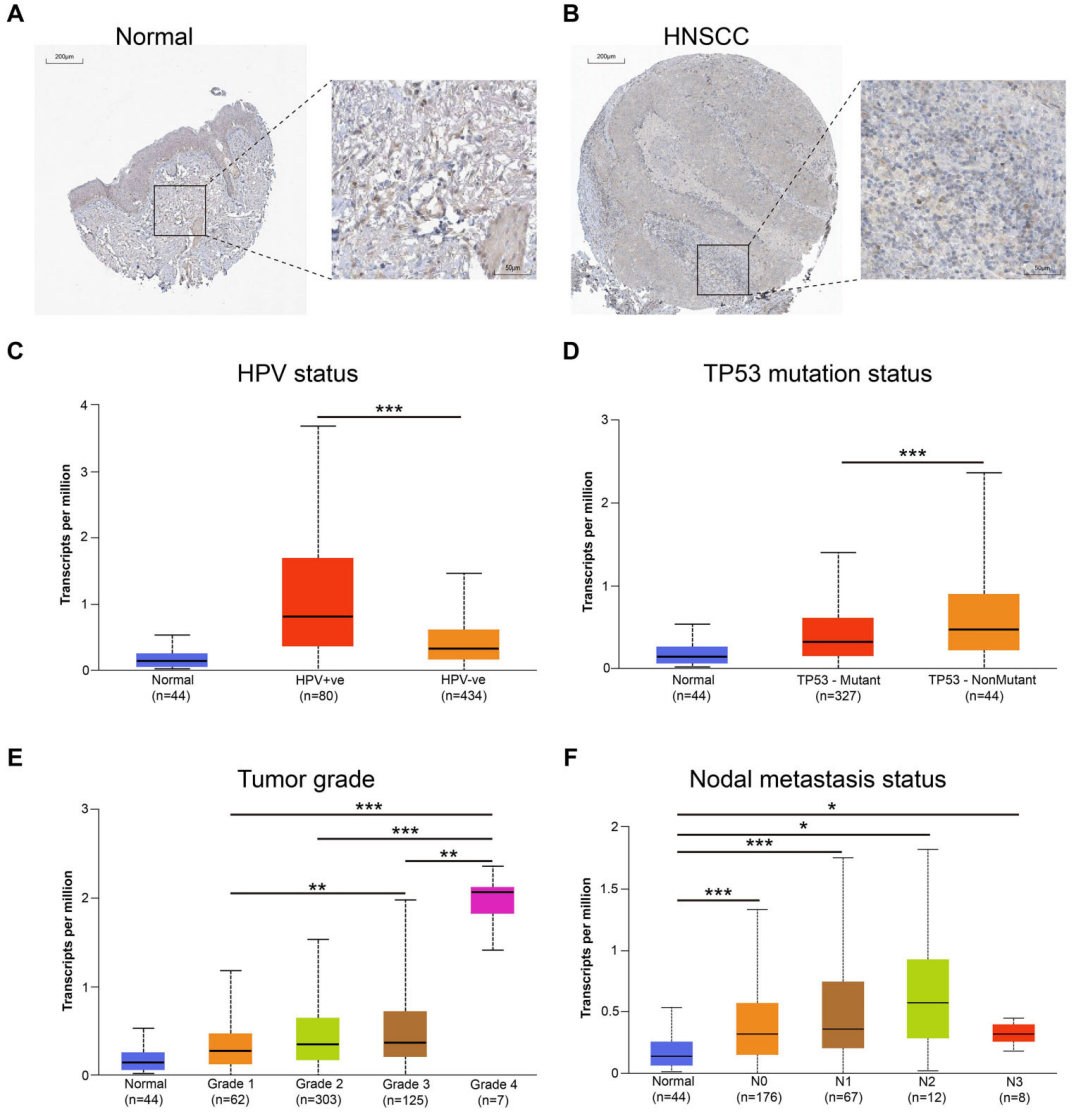
ZC3H12D immunohistochemical staining in normal oral samples and head and neck squamous cell carcinoma (HNSCC) samples from the HPA database (**A** and **B**, scale bar 200 μm; enlargement scale bar_50_ μm). Box plots using the UALCAN database that assessed ZC3H12D expression in various patient subgroups based on clinical variables: HPV status (**C**), TP53 mutation status (**D**), tumor grade (**E**), and nodal metastasis status (**F**). Data are reported as median and IQR. *P<0.05, **P<0.01, ***P<0.001 (Pearson's chi-squared test). +ve and -ve indicate positive and negative for HPV, respectively.

**Figure 4:**
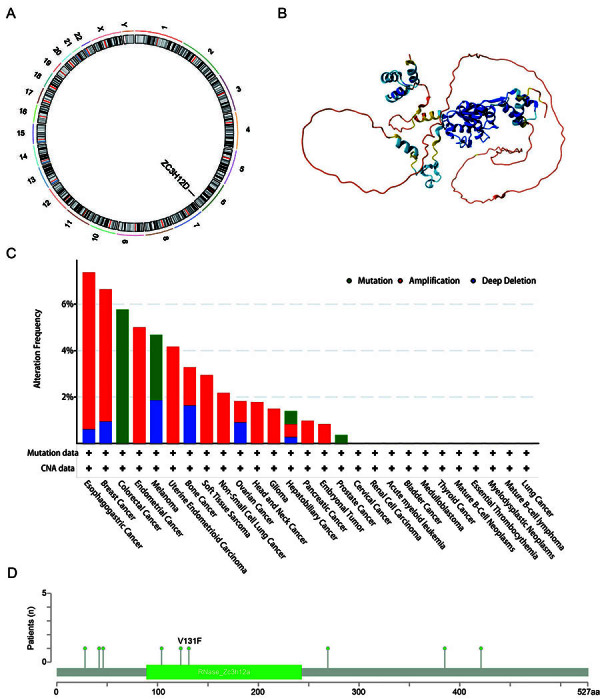
**A**, Circular visualization of the chromosome location of ZC3H12D. **B**, Three-dimensional structure of ZC3H12D protein. **C**, Genetic alteration features of ZC3H12D in a variety of cancers. **D**, ZC3H12D mutation sites displayed in the lollipop diagram.

As shown in Figure 3A and B, normal oral mucosa tissue has low expression of ZC3H12D protein, while HNSCC tissues have high expression of ZC3H12D protein. The clinicopathological characteristics of 497 HNSCC samples from TCGA are listed in Table 1. Depending on the level of ZC3H12D expression, patients with HNSCC were split into two groups (low/high). UALCAN database was employed to evaluate the variations in the expression of ZC3H12D between the clinical subgroups of the normal and HNSCC samples. Figure 3C-F demonstrates that various subgroups of patients with HNSCC had significantly higher expression levels of ZC3H12D, including tumor grade, TP53 mutation status, nodal metastasis status, and HPV status subgroups. As shown in Figure 4A, ZC3H12D is located on chromosome 6. Figure 4B shows the three-dimensional structure of the ZC3H12D protein. Furthermore, we examined the mutational characterization of ZC3H12D in a variety of cancer types, with “amplification” being the main type of change in HNSCC (Figure 4C). This may account for the overexpression of ZC3H12D in HNSCC. The sites as well as the corresponding domain of ZC3H12D mutations are also displayed (Figure 4D).

**Table 1:** Relationship between clinicopathological characteristics and ZC3H12D expression in patients with head and neck squamous cell carcinoma (HNSCC).

Characteristics	Low expression of ZC3H12D	High expression of ZC3H12D	P
n	249	248	
Age, n (%)			0.280
>60	121 (24.4%)	133 (26.8%)	
≤60	128 (25.8%)	114 (23%)	
Gender, n (%)			0.461
Female	62 (12.5%)	70 (14.1%)	
Male	187 (37.6%)	178 (35.8%)	
Grade, n (%)			0.243
G1	32 (6.7%)	29 (6.1%)	
G2	156 (32.7%)	140 (29.4%)	
G3	53 (11.1%)	65 (13.6%)	
G4	0 (0%)	2 (0.4%)	
Stage, n (%)			0.061
Stage I	10 (2.1%)	9 (1.9%)	
Stage II	50 (10.4%)	45 (9.3%)	
Stage III	61 (12.6%)	41 (8.5%)	
Stage IV	119 (24.6%)	148 (30.6%)	
T, n (%)			0.419
T1	16 (3.3%)	17 (3.5%)	
T2	64 (13.3%)	80 (16.6%)	
T3	70 (14.5%)	59 (12.2%)	
T4	90 (18.7%)	86 (17.8%)	
N, n (%)			0.061
N0	129 (27.2%)	110 (23.2%)	
N1	42 (8.8%)	38 (8%)	
N2	61 (12.8%)	90 (18.9%)	
N3	2 (0.4%)	3 (0.6%)	
M, n (%)			0.358
M0	230 (48.7%)	237 (50.2%)	
M1	4 (0.8%)	1 (0.2%)	

Pearson's chi-squared test.

### Prognostic value of ZC3H12D in HNSCC

**Figure 5:**
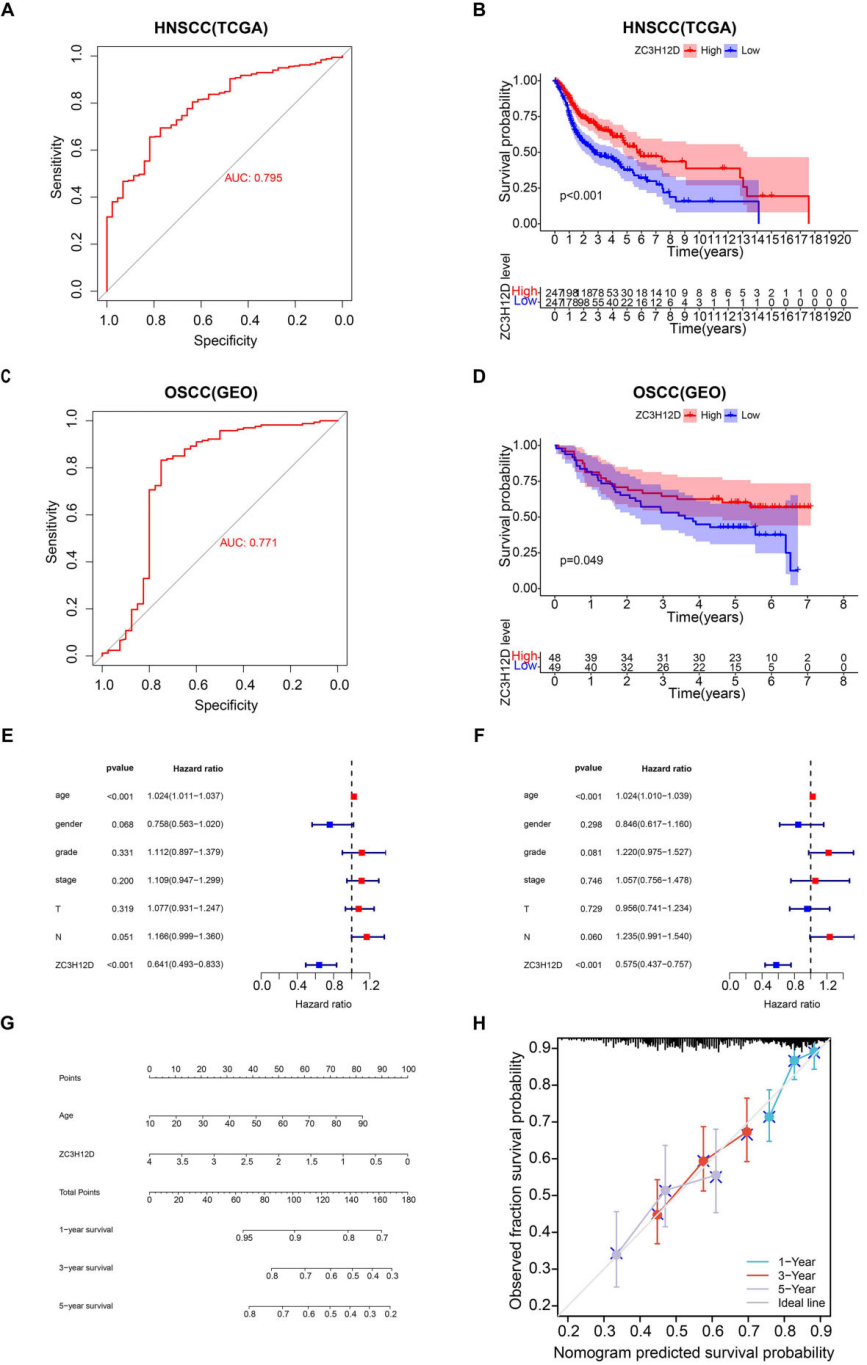
High ZC3H12D expression was related to a better prognosis in head and neck squamous cell carcinoma (HNSCC) patients. **A** and **C**, ROC curve analysis of ZC3H12D for the diagnosis of HNSCC patients and oral squamous cell carcinoma (OSCC) patients. **B** and **D**, The Kaplan-Meier overall survival analysis of HNSCC patients and OSCC patients from TCGA and GEO databases. **E**, Univariate and (**F**) multivariate analyses of clinicopathologic features and survival in HNSCC patients from TCGA. Nomogram model for HNSCC patients (**G**) and the calibration curve of the nomogram model (**H**) for predicting survival status at 1, 3, and 5 years.

The ability of ZC3H12D to distinguish between normal and tumor samples was evaluated via the ROC curve. As illustrated in Figure 5A and C, the AUC value was up to 0.795 in HNSCC and up to 0.771 in OSCC, which indicated the significant diagnostic value of ZC3H12D for HNSCC patients and OSCC patients. In addition, ZC3H12D was verified to have significant diagnostic value for OSCC patients by data from TCGA (Supplementary Figure S1C). The K-M curve of overall survival was employed for survival analysis of the TCGA-HNSC data set. The findings demonstrated that HNSCC patients who expressed low levels of ZC3H12D had shorter survival times than those who expressed high levels of ZC3H12D (P<0.001) (Figure 5B), suggesting a correlation between high ZC3H12D expression and better prognosis for HNSCC patients. Further analysis showed that these results were in line with the outcomes of GEPIA2 (Supplementary Figure S2), and that patients with high expression of ZC3H12D showed superior overall outcomes (P<0.001, HR=0.6). The clinical data obtained from GSE41613 dataset showed that OSCC patients with high ZC3H12D expression had longer survival time and better prognosis (Figure 5D). The TCGA data also suggested that OSCC patients with high ZC3H12D expression had better prognosis (Supplementary Figure S1D). Age and ZC3H12D expression were shown to be strongly linked with overall survival using univariate Cox analysis, and multivariate Cox analysis was employed to further identify ZC3H12D expression and age as the independent prognostic factors in patients with HNSCC (P<0.001) (Figure 5E and F). Lastly, the nomogram model was constructed to forecast the 1-, 3-, and 5-year overall survival (OS) of patients (Figure 5G). The calibration curve demonstrated that the model's prediction and the actual observation agreed rather well (Figure 5H).

### GO and KEGG enrichment analysis of ZC3H12D and its co-expressed genes in HNSCC

**Figure 6:**
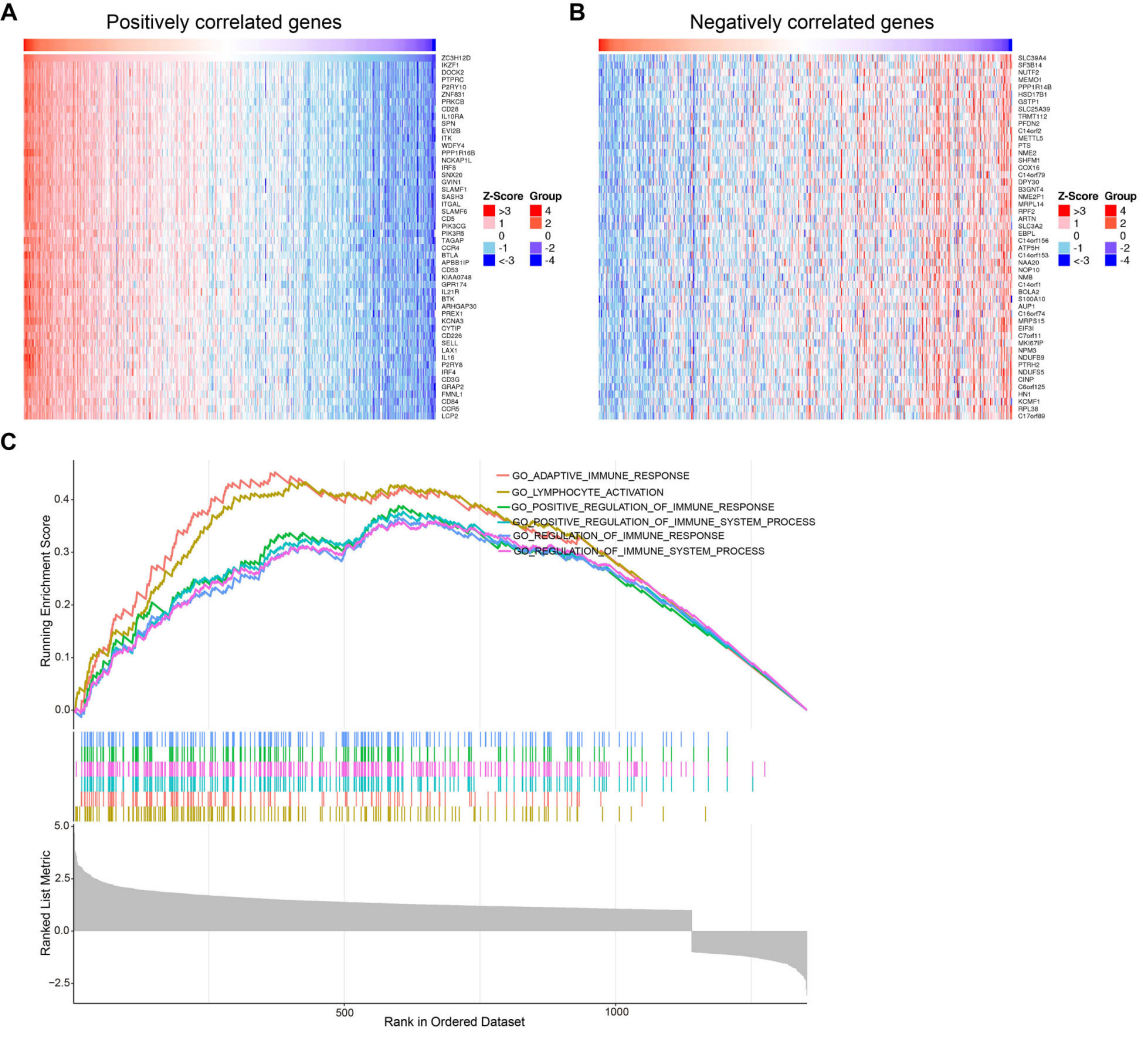
Functional enrichment analyses of ZC3H12D. **A**, Heatmap of the top 50 genes positively correlated with ZC3H12D in head and neck squamous cell carcinoma (HNSCC). **B**, Heatmap of the top 50 genes negatively correlated with ZC3H12D in HNSCC. **C**, Enrichment results of Gene Set Enrichment Analysis (GSEA).

**Figure 7:**
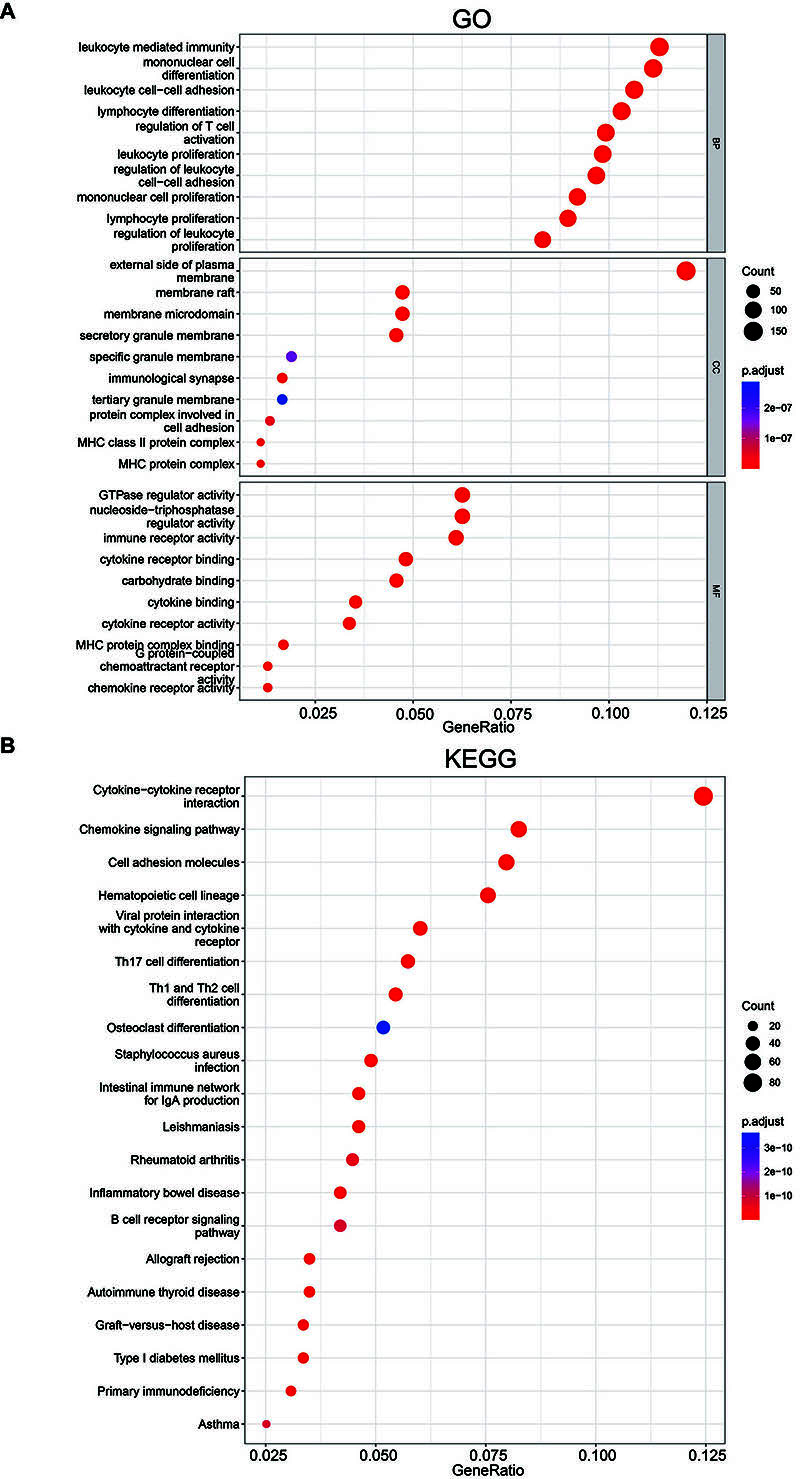
**A**, GO enrichment analysis of ZC3H12D-coexpressed genes. **B**, KEGG enrichment analysis of ZC3H12D-coexpressed genes.

To investigate the co-expression profile of ZC3H12D in HNSCC, the LinkedOmics database was employed, and 1445 genes were identified to be co-expressed with ZC3H12D (FDR <0.05, |cor| >0.4). The top 50 genes in HNSCC that were positively or negatively correlated with ZC3H12D are displayed in Figure 6A and B. GO and KEGG enrichment analysis was performed using co-expressed genes of ZC3H12D to explore ZC3H12D-related pathways and biological functions. These co-expressed genes were primarily enriched in leukocyte mediated immunity, mononuclear cell differentiation, leukocyte cell-cell adhesion, and lymphocyte differentiation under the BP category, while the co-expressed genes were mainly involved in the external side of the plasma membrane under the CC category. Moreover, the co-expressed genes were primarily enriched in GTPase regulator activity and immune receptor activity under the MF category (Figure 7A). In addition, Figure 7B displays the top 20 KEGG enriched pathways for ZC3H12D and its co-expressed genes. KEGG enrichment analysis revealed the main enrichment in cell adhesion molecules, chemokine signaling pathway, and cytokine-cytokine receptor interaction. These results suggested that ZC3H12D may play a role in HNSCC's immune response and influence the effectiveness of immunotherapy through a variety of mechanisms.

### GSEA identified ZC3H12D-related signaling pathways

GSEA was conducted to further explore the underlying molecular mechanisms impacted by ZC3H12D in HNSCC. Out of 178 signaling pathways, 155 were up-regulated and 54 were significantly enriched at P<0.001 and q-value <0.001 (Supplementary Table S3). The significantly up-regulated pathways involved in immune response include ‘Adaptive immune response', ‘Lymphocyte activation', ‘Positive regulation of immune response', ‘Positive regulation of immune system process', ‘Regulation of immune response', and ‘Regulation of immune system process (Figure 6C). These findings indicated that ZC3H12D plays a role in HNSCC immune response regulation.

### Immune characteristics analysis of ZC3H12D in HNSCC

**Figure 8:**
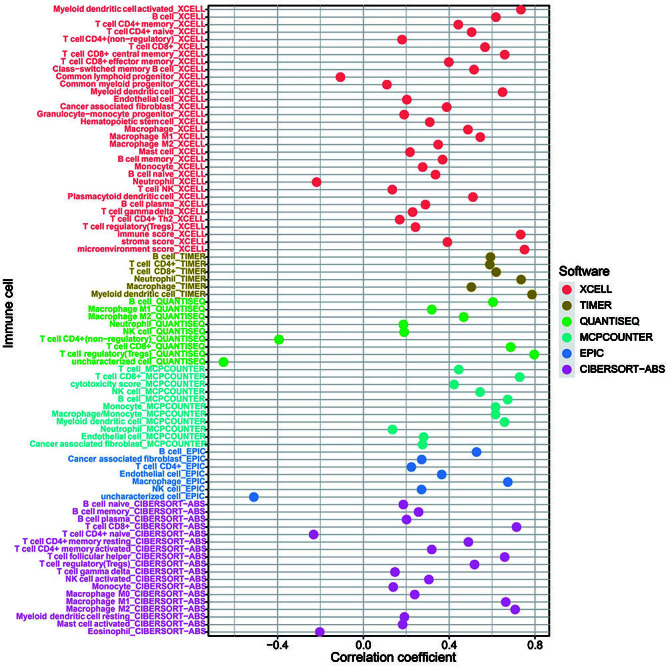
The majority of immune cells were significantly positively correlated with ZC3H12D expression.

**Figure 9:**
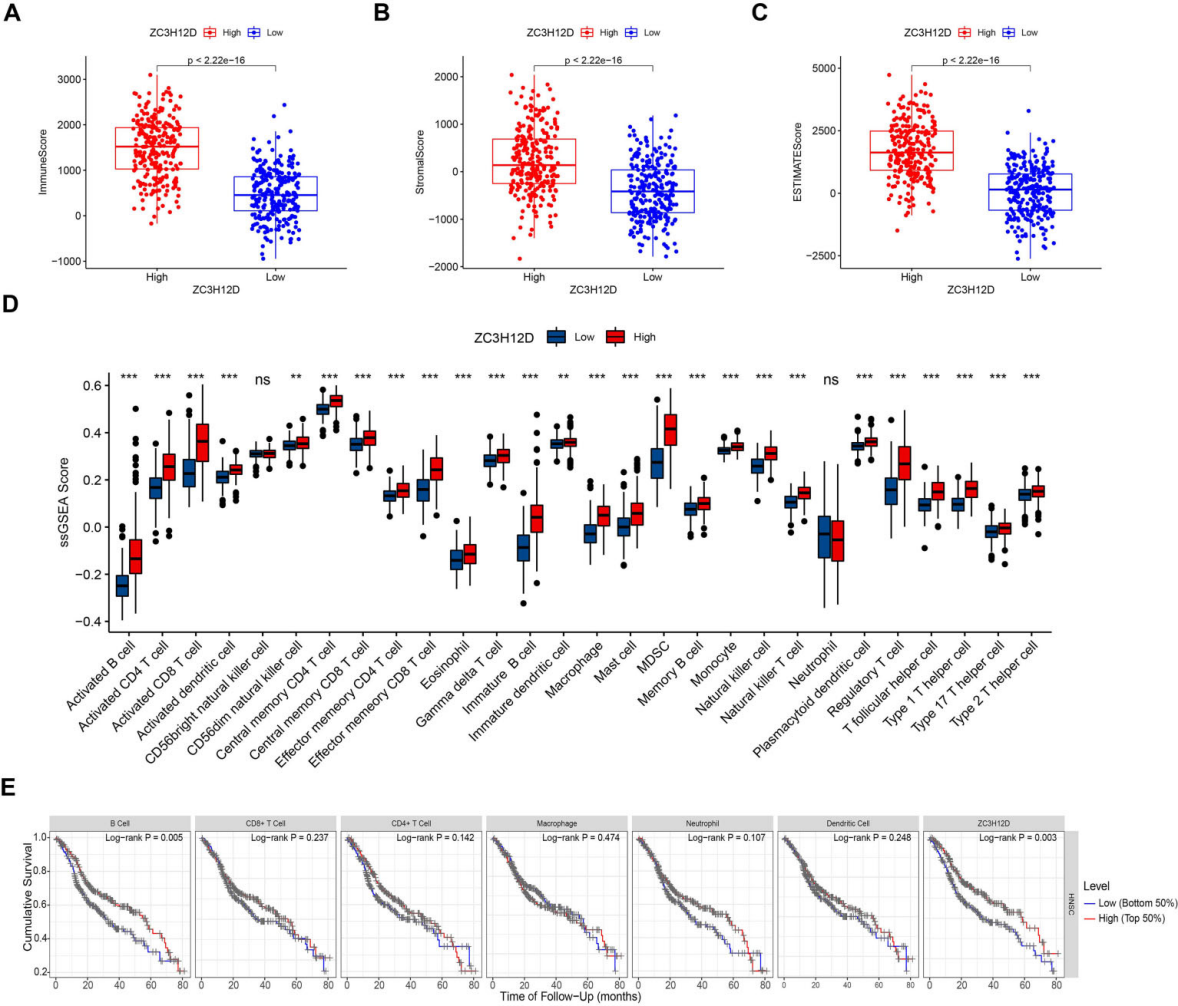
Immune infiltration analysis. **A**-**D**, The high ZC3H12D expression group showed higher tumor microenvironment (TME) scores and ssGSEA scores. Data are reported as median and IQR. Wilcoxon rank-sum test. **E**, Correlation between abundance of immune infiltration and survival in head and neck squamous cell carcinoma (HNSCC). ns: non-significant.

The immune cell infiltration in HNSCC patients was determined to better understand the ZC3H12D effect on the TME. The findings showed that ZC3H12D expression was positively correlated with most of the immune cells, including macrophage (r=0.673, P<0.001), CD8+ T cell (r=0.729, P<0.001), NK cell (r=0.544, P<0.001), T cell regulatory (r=0.796, P<0.001), B cell (r=0.673, P<0.001), and CD4+ T cells (r=0.589, P<0.001) (Figure 8, Supplementary Table S4). To assess the distribution of stromal and immune cells in the TME, we calculated the Immune/Stromal/ESTIMATE score using the ESTIMATE algorithm. Compared to the group with low expression of ZC3H12D, the group with high expression had a markedly higher stromal score, ESTIMATE score, and immune score (Figure 9A-C). In addition, ssGSEA was employed to quantify immune cell infiltration of HNSCC samples in the TCGA database, and the groups with low and high ZC3H12D expression were compared (Figure 9D). The ssGSEA results further indicated that patients with higher ZC3H12D expression may experience more active immune responses. TIMER showed that B cell infiltration and ZC3H12D expression were related to survival of HNSCC patients, and patients with low levels of B cell infiltration and low ZC3H12D expression had a poorer prognosis (Figure 9E). Furthermore, TIMER2 showed that HNSCC patients with high levels of CD4+ and CD8+ T cell infiltration had a better prognosis at high ZC3H12D expression levels, and high B cell infiltration levels corresponded to a better prognosis at consistent ZC3H12D expression levels (Supplementary Figure S3A-C).

**Figure 10:**
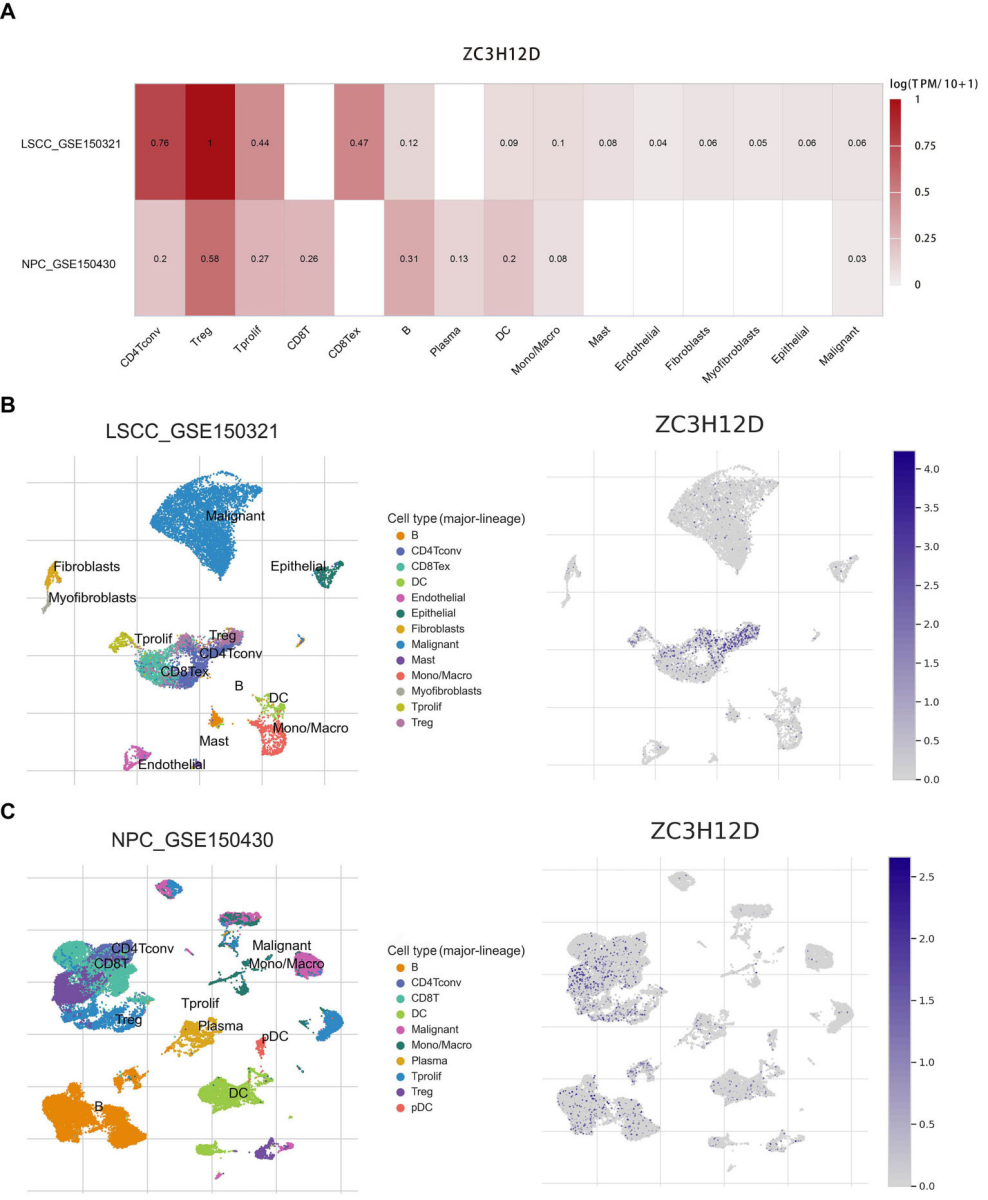
ZC3H12D expression distribution in various cell types in the tumor microenvironment as determined by single-cell transcriptome analysis. **A**, Correlation between ZC3H12D expression and various cell types in the GSE150321 and GSE150430 datasets. **B** and **C**, ZC3H12D expression status in various cell types in the GSE150321 and GSE150430 datasets. LSCC: laryngeal squamous cell carcinoma; NPC: nasopharyngeal carcinoma.

Furthermore, TIMER2 database was used to examine the correlation between the expression of the major immune checkpoint genes and ZC3H12D expression, including *SIGLEC15*, *TIGIT*, *HAVCR2*, *CTLA4*, *PDCD1*, *PDCD1LG2*, *CD274*, and *LAG3*. Supplementary Figure S4A and B showed that the expression of the above immune checkpoint genes was strongly positively correlated with ZC3H12D expression in HNSCC. Moreover, the expression of all eight major immune checkpoint genes was upregulated in HNSCC patients with high ZC3H12D expression (Supplementary Figure S4C). Lastly, analysis of single-cell RNA sequencing data based on the TISCH2 database revealed that ZC3H12D expression was distributed across multiple immune cell types in the tumor microenvironment of HNSCC (Figure 10A-C).

## Discussion

HNSCC is a type of squamous cell tumor that can occur in any area of the head or neck (13). Despite ongoing advancements in HNSCC treatment methods, the survival rate has not improved significantly (30). To increase patient survival, it is crucial to find new genes connected to the occurrence and development of HNSCC. Greater treatment sensitivity and specificity are made possible by the identification of new targeted genes. The improvement of treatment outcomes of HNSCC particularly depends on the discovery of new molecular targets relating to immune infiltration. Here, we investigated the role played by the ZC3H12D gene in HNSCC and how it affects tumor immune infiltration.

In this study, we first investigated the expression of ZC3H12D in HNSCC and in various tumors under HNSCC classification. The results showed that ZC3H12D expression was significantly increased in HNSCC compared with normal tissues. In different tumors under the classification of HNSCC, the ZC3H12D expression in OSCC was significantly higher than that in normal tissues. A previous study revealed that the methylation level of the ZC3H12D promoter in HNSCC patients was lower than that in normal patients (10), and our study showed that “amplification” was the main type of ZC3H12D gene alteration in HNSCC. These could be potential explanations for the overexpression of ZC3H12D in HNSCC. Furthermore, expression and prognostic analyses of ZC3H12D indicated that ZC3H12D could serve as a potential diagnostic and prognostic biomarker for HNSCC, especially OSCC under the classification of HNSCC, and that increased expression of ZC3H12D was associated with better clinical outcomes. Prior research has shown that by inhibiting RB1 phosphorylation, ZC3H12D overexpression inhibits the progression from G1 to S phase and thus regulates cell growth (31). Tomita et al. (32) reported that ZC3H12D enhanced tumoricidal activity by capturing extracellular nex-interleukin-1β (IL1β)-mRNA. These mechanisms of action may be potential explanations for why HNSCC patients with higher ZC3H12D expression have better clinical outcomes. In addition, KEGG and GO analysis results showed that ZC3H12D might be involved in leukocyte mediated immunity, lymphocyte differentiation, and chemokine signaling pathway. GSEA analysis results demonstrated that ZC3H12D was mainly enriched in functions such as adaptive immune response and lymphocyte activation. These findings indicated that ZC3H12D may actively participate in immune defense and tumor surveillance in HNSCC, thereby inhibiting tumor progression.

Advanced HNSCC patients receiving immune checkpoint blockade therapy have a distinct survival advantage, according to recent clinical trials. Recent findings demonstrate that HNSCC is one of the most hopeful cutting-edge fields in immunotherapy research (33). Immune infiltrating cells are essential to the TME and have a significant influence on the response and prognosis of immunotherapy (34). In this study, ZC3H12D expression was distributed across a variety of immune cell types in the TME of HNSCC based on TISCH2 analysis of single-cell RNA sequencing data. Results might differ depending on the methods used in immune infiltration calculation, such as different marker genes and different algorithms. Examples are the positive correlation between neutrophil infiltration and ZC3H12D according to the TIMER method and the negative correlation according to CIBERSORT and XCELL (35). However, all calculation methods showed that ZC3H12D expression was positively correlated with the infiltration of most immune cells, including macrophages, CD8+ T cells, CD4+ T cells, and B cells. This suggested that patients with high ZC3H12D expression might present with relatively high populations of these immune cells. Increased levels of CD8+ and CD4+ T cell infiltration in tumor tissues of HNSCC are related to a better prognosis (36). CD8+ T cells are crucial effector cells in tumor immunity. (37). B cells are considered to be involved in the tumor-promoting and anti-tumor immunity of HNSCC (38). According to reports, macrophages are associated to tumor invasion and growth, while being especially abundant in the TME of HNSCC (39). Therefore, we hypothesized that the survival difference between patients with low and high ZC3H12D expression may be caused by differences of infiltrating immune cell subtypes and proportions. In addition, the findings demonstrated that the ZC3H12D high-expression group had higher TME scores, indicating ZC3H12D might be implicated in changing the TME of HNSCC. Moreover, we found that the expression of the main immune checkpoint genes, such as *PDCD1*, *CTLA4*, *TIGIT*, and *HAVCR2*, positively correlated with ZC3H12D expression and was upregulated in HNSCC patients with high ZC3H12D expression, indicating that patients with high ZC3H12D expression may respond to immunotherapy better. These results consistently demonstrated the close relationship between ZC3H12D expression and tumor immunity in HNSCC.

Although this study advances our understanding of the relationship between ZC3H12D and HNSCC, it still has some limitations. Firstly, since the data were sourced from open databases, the quality of the data cannot be determined. To validate our findings, additional experimental studies are required. Secondly, the majority of analyses in the present research were based on the mRNA levels of ZC3H12D. More in-depth study based on protein levels would provide more convincing findings. Moreover, gene-based markers are inadequate as biometric characteristics or prognostic models for the prediction of patient outcomes. For prediction to be more accurate and useful, subnetwork or network markers must be found. According to reports, an approach that can discover novel survival prognostic subnetwork signatures was created to explore the association between clinical metastatic potential, representative protein-protein interactions, and gene expression patterns (40). Finally, in order to address the changes in the TME under diverse therapy plans, investigations at the single-cell level are needed.

## Conclusions

In summary, the present study identified ZC3H12D as a potential prognostic biomarker for HNSCC, especially OSCC. In addition, the findings showed that ZC3H12D closely correlated with immune infiltration in HNSCC, which is helpful for immunotherapy against HNSCC.

## Supplementary Material

Click to view [zip].
