# Supplementary material for: ZC3H12D upregulation in head and neck squamous cell carcinoma: a potential prognostic biomarker associated with immune infiltration
Source: Braz J Med Biol Res. 2025 Feb 3;58:e14227. doi: 10.1590/1414-431X2024e14227 (PMC11793147; doi:10.1590/1414-431X2024e14227)

Figure S1. A, Expression of ZC3H12D in normal and oral squamous cell carcinoma (OSCC) samples based on data from TCGA. **B**, Expression of ZC3H12D in normal and laryngeal squamous cell carcinoma (LSCC) samples based on data from TCGA. **C**, ROC curve analysis of ZC3H12D for the diagnosis of OSCC patients based on data from TCGA. **D**, The Kaplan-Meier overall survival analysis of OSCC patients from TCGA. Data are reported as median and IQR. Wilcoxon rank-sum test.

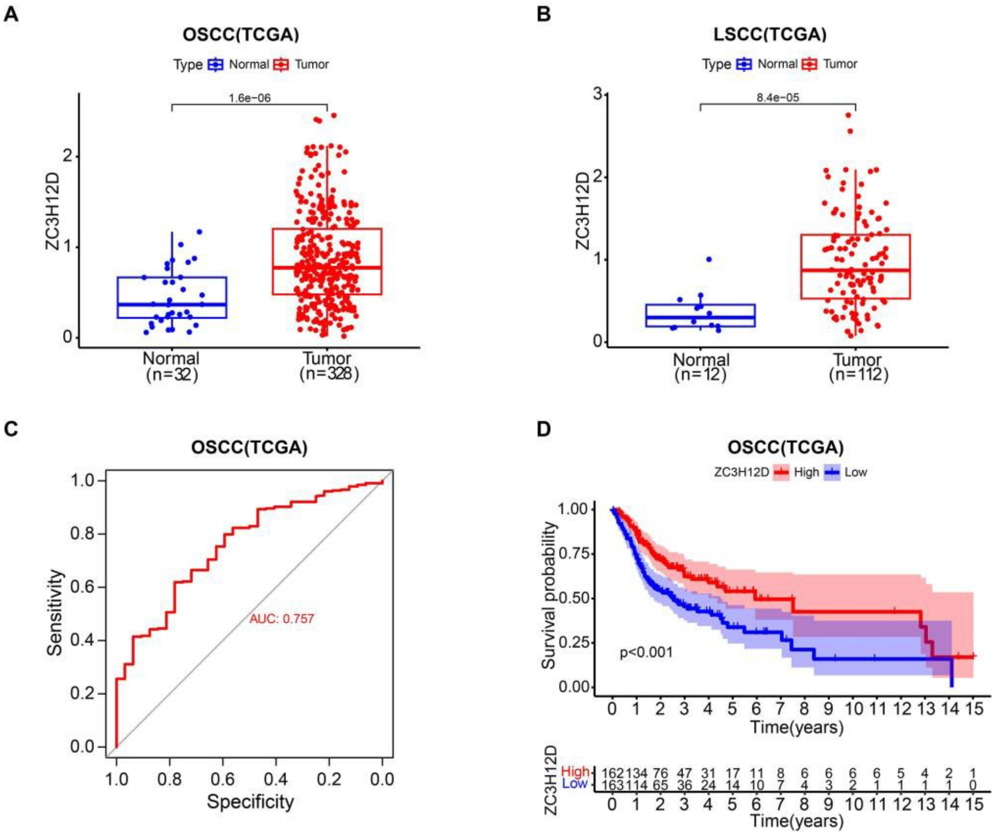

Figure S2. Overall survival curves of TCGA patients by GEPIA2.

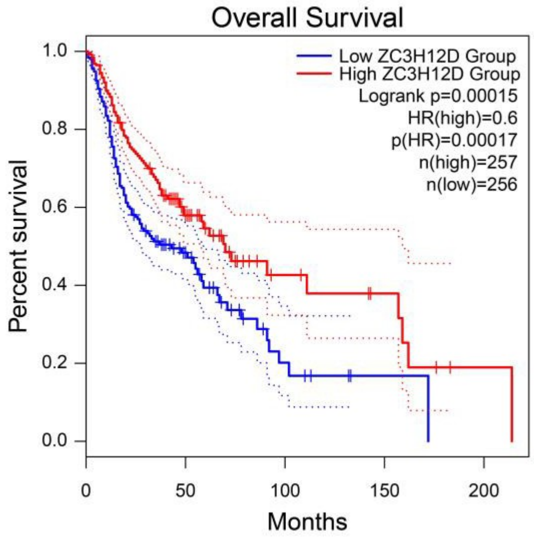

Figure S3. A–C, Kaplan-Meier survival curves based on the levels of CD4+ and CD8+ T cell infiltration and B cell infiltration in head and neck squamous cell carcinoma patients with high and low ZC3H12D expression.

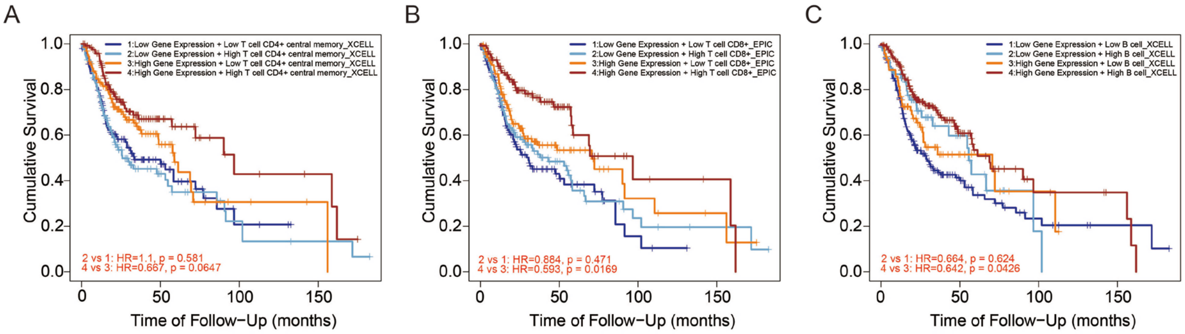

Figure S4. Analysis of the correlation between checkpoint-related genes and ZC3H12D expression in pan-cancer (**A**). Correlations of ZC3H12D expression with the main immune checkpoints in head and neck squamous cell carcinoma (HNSCC) (**B**). Comparison of immune checkpoints expression between the groups with high and low ZC3H12D expression in HNSCC patients (**C**). Data are reported as median and IQR. ***P<0.001, Wilcoxon rank-sum test.

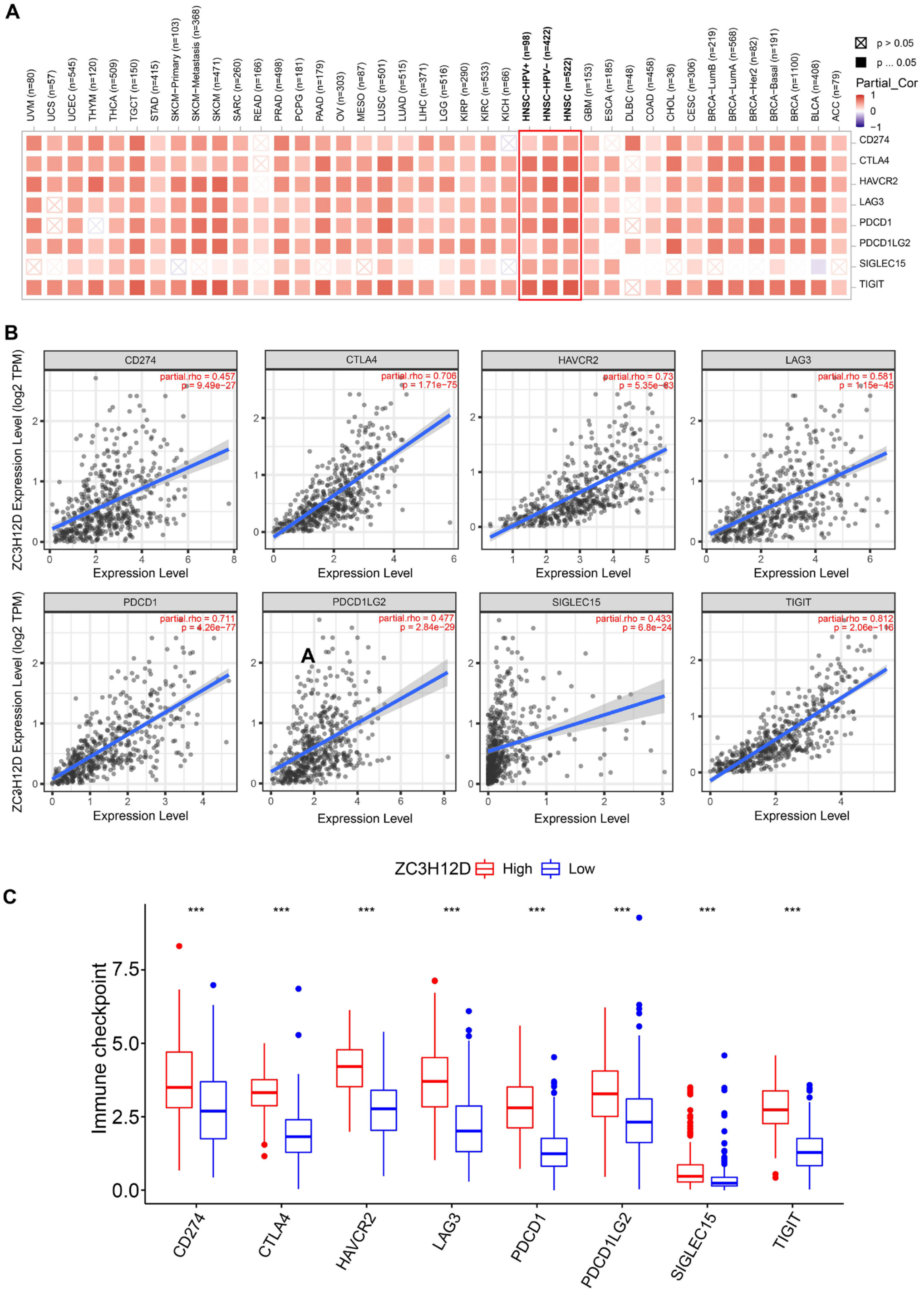

Supplement: Supplementary file 1 [file 1414-431X-bjmbr-58-e14227-suppl.zip › 14227_Supplementary Figures.pdf]
